# Phase II study of long‐course chemoradiotherapy followed by consolidation chemotherapy as total neoadjuvant therapy in locally advanced rectal cancer in Japan: ENSEMBLE‐2

**DOI:** 10.1002/ags3.12848

**Published:** 2024-08-03

**Authors:** Yoshinori Kagawa, Koji Ando, Mamoru Uemura, Jun Watanabe, Koji Oba, Yasunori Emi, Nobuhisa Matsuhashi, Naoki Izawa, Osamu Muto, Tatsuya Kinjo, Ichiro Takemasa, Eiji Oki

**Affiliations:** ^1^ Department of Gastroenterological Surgery Osaka General Medical Center Osaka Japan; ^2^ Department of Gastroenterological Surgery Osaka International Cancer Institute Osaka Japan; ^3^ Department of Surgery and Science Kyushu University Fukuoka Japan; ^4^ Department of Gastroenterological Surgery Osaka University Osaka Japan; ^5^ Department of Surgery, Gastroenterological Center Yokohama City University Medical Center Yokohama Japan; ^6^ Department of Colorectal Surgery Kansai Medical University Osaka Japan; ^7^ Department of Biostatistics Graduate School of Medicine, the University of Tokyo Tokyo Japan; ^8^ Department of Surgery Saiseikai Fukuoka General Hospital Fukuoka Japan; ^9^ Department of Gastroenterological and Pediatric Surgery Gifu University Graduate School of Medicine Gifu Japan; ^10^ Department of Clinical Oncology St. Marianna University School of Medicine Kawasaki Japan; ^11^ Department of Clinical Oncology Akita Redcross Hospital Akita Japan; ^12^ Department of Digestive and General Surgery, Faculty of Medicine University of the Ryukyu Okinawa Japan; ^13^ Department of Surgery, Surgical Oncology, and Science Sapporo Medical University School of Medicine Sapporo Japan

**Keywords:** locally advanced rectal cancer, nonoperative management, pathological complete response, total mesorectal excision, total neoadjuvant therapy

## Abstract

**Aim:**

To evaluate the feasibility and safety of total neoadjuvant therapy with long‐course chemoradiotherapy followed by consolidation chemotherapy in Japanese patients with locally advanced rectal cancer.

**Methods:**

This prospective, multicenter, single‐arm, phase II trial was conducted at 10 centers. The eligibility criteria included age ≥20 y, locally advanced rectal cancer within 12 cm of the anal verge, and cT3‐4N0M or TanyN+M0 at diagnosis, enabling curative resection. The protocol treatment was capecitabine (1650 mg/m^2^/day)‐based long‐course chemoradiotherapy (50.4 Gy/28 fractions) and consolidation chemotherapy (CAPOX, four courses) followed by total mesorectal excision. Nonoperative management was allowed if a clinical complete response was achieved. The primary endpoint was the pathologic complete response rate.

**Results:**

Among 28 enrolled patients (19 men, 9 women; median age, 69.5 [41–79] y), the long‐course chemoradiotherapy and consolidation chemotherapy completion rates were 100% and 96.4%, respectively. The clinical responses included clinical complete response, (35.7%, 10/28), near‐complete response (28.6%, 8/28), and incomplete response (32.1%, 9/28). Total mesorectal excision and nonoperative management were performed in 21 and six patients, respectively. The final analysis included 21 patients. Five patients (23.8% [90% confidence interval 11.8%–41.8%]) achieved pathologic complete response, while 10 of 28 patients (35.7%) achieved a pathological complete response or a sustained clinical complete response. No treatment‐related deaths occurred. Grade ≥3 adverse events included diarrhea (7.1%) and leukopenia (7.1%).

**Conclusion:**

ENSEMBLE‐2 demonstrated comparable pathologic complete response rates and well‐tolerated safety of total neoadjuvant therapy with long‐course chemoradiotherapy followed by consolidation chemotherapy in Japanese patients with locally advanced rectal cancer.

## Graphical abstract

ENSEMBLE‐2 was a multicenter, single‐arm phase II clinical trial investigating the safety and efficacy of total neoadjuvant therapy for patients with locally advanced rectal cancer in Japan. The key eligibility criteria were age 20 y or older, locally advanced rectal cancer within 12 cm from the anal verge, and cT3‐4N0M0 or TanyN+M0 that was curatively resectable at diagnosis. Long‐course chemoradiotherapy was administered at 1.8 Gy per day over 28 d for a total dose of 50.4 Gy. Consolidative chemotherapy was initiated 14 ± 7 d after LCCRT. Surgery was performed with curative intent 21–56 d after consolidative chemotherapy. Additional lateral lymph node dissection was performed at the surgeon's discretion. The primary endpoint was the pathological complete rate.

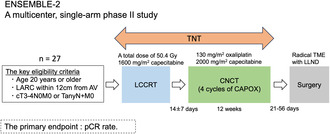

## 1 INTRODUCTION

In 2023, ~800,000 new patients with rectal cancer will be diagnosed worldwide, half of which will be locally advanced rectal cancer (LARC), defined as stage II (cT3 or cT4 and N0) or stage III (cTany and N+) rectal cancer.^1^

Although multimodal treatment strategies, including total mesorectal excision (TME), preoperative chemoradiotherapy (CRT), lateral lymph node dissection (LLND), and postoperative adjuvant chemotherapy (ACT),^2^, ^3^, ^4^ have improved LARC outcomes, the mortality rate of LARC has improved only slightly in the past decade because of the high rate of distant metastases (29%–39%).^4^, ^5^, ^6^, ^7^, ^8^

In recent years, a novel therapeutic approach, total neoadjuvant therapy (TNT), has been developed in Europe, the United States, and Asia to enhance the long‐term prognosis of LARC compared with conventional CRT.^9^, ^10^, ^11^, ^12^, ^13^ This approach entails the sequential administration of preoperative chemotherapy and chemoradiotherapy. However, Japanese guidelines recommend upfront surgery and ACT for LARC, with LLND as an optional procedure.^14^ To date, no prospective multicenter clinical trials in Japan have evaluated TNT for patients with LARC, except for ENSEMBLE‐1 (jRCT s051200113), which investigated TNT consisting of short‐course radiotherapy (SCRT) followed by six cycles of CAPOX.^15^

Therefore, we conducted a prospective, multicenter, single‐arm study (ENSEMBLE‐2) to evaluate the feasibility and safety of TNT with long‐course CRT (LCCRT) followed by consolidation chemotherapy (CNCT) consisting of four cycles of CAPOX for patients with LARC in Japan.

## 2 METHODS

### 2.1 Trial design and participants

**FIGURE 1:**
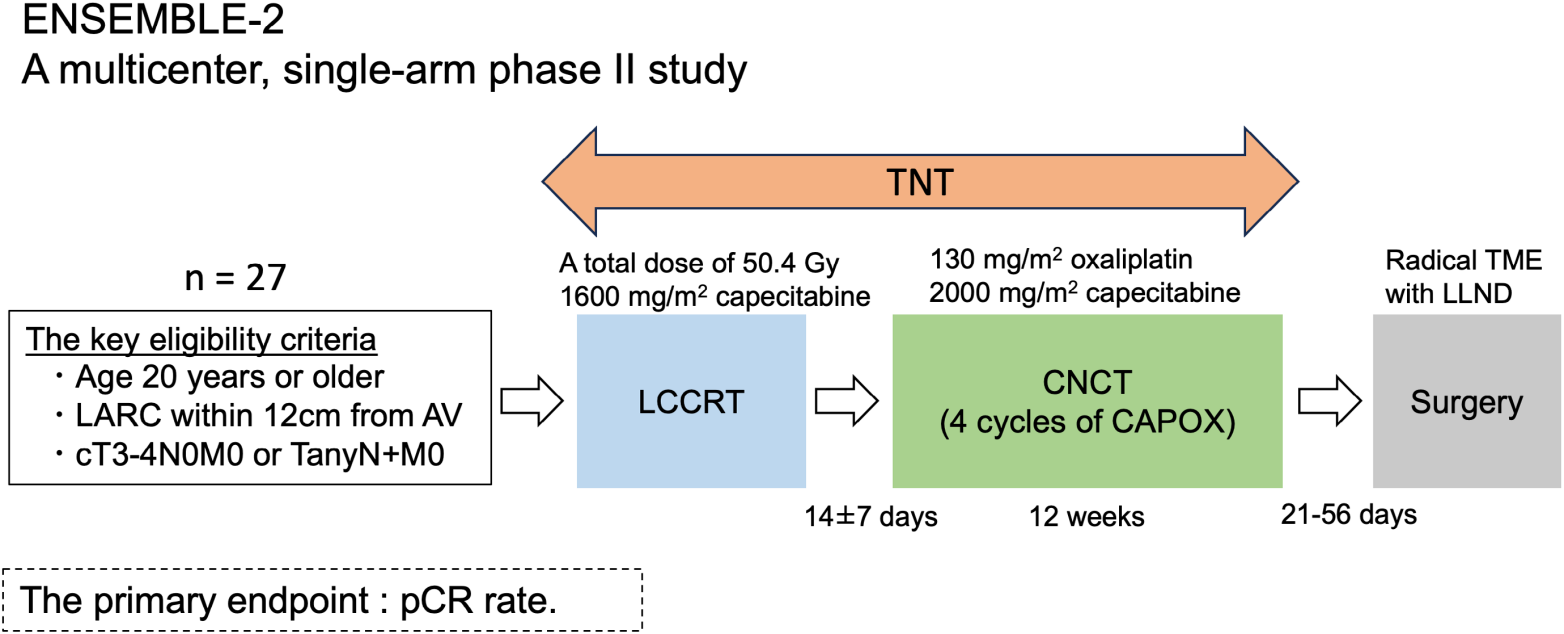
Study design. ENSEMBLE‐2 was a multicenter, single‐arm phase II clinical trial investigating the safety and efficacy of total neoadjuvant therapy for patients with locally advanced rectal cancer (LARC) in Japan. The key eligibility criteria were age 20 y or older, locally advanced rectal cancer within 12 cm from the anal verge (AV), and cT3‐4N0M0 or TanyN+M0 that was curatively resectable at diagnosis. Long‐course chemoradiotherapy (LCCRT) was administered at 1.8 Gy per day over 28 d for a total dose of 50.4 Gy. Consolidative chemotherapy (CNCT) was initiated 14 ± 7 d after LCCRT. CAPOX consisted of an intravenous infusion of 130 mg/m^2^ oxaliplatin on Day 1, along with oral administration of 2000 mg/m^2^ capecitabine on Days 1–14, repeated every 3 weeks for a total of four cycles. Surgery was performed with curative intent 21–56 d after CNCT. Additional lateral lymph node dissection (LLND) was performed at the surgeon's discretion. The primary endpoint was the pathological complete (pCR) rate. LARC, locally advanced rectal cancer; AV, anal verge; LCCRT, long‐course chemoradiotherapy; TME, total mesorectal excision; LLND, lateral lymph node dissection; pCR, pathological complete response.

The ENSEMBLE‐2 study is a prospective, multicenter, open‐label, single‐arm, phase II trial conducted at 10 institutions (Figure 1). Before study commencement, the protocol was reviewed and approved by the Clinical Research Review Board of Kyushu University (ID: CRB7180005) and the Institutional Review Board of each participating hospital. All patients provided written informed consent before enrollment in the study. This study was registered in the Japan Registry of Clinical Trials (jRCTs071210143).

The eligibility criteria were (a) written informed consent; (b) a histological diagnosis of primary rectal adenocarcinoma; (c) no distant metastases on computed tomography (CT) or positron emission tomography (PET) and radical resection was clinically possible; (d) age ≥20 y; (e) Eastern Cooperative Oncology Group Performance status (ECOG‐PS) of 0 or 1 (ECOG‐PS 0 for age ≥71 y); (f) no prior treatment for rectal cancer; (g) lower margin of the tumor within 12 cm from the anal verge (AV); (h) clinically diagnosed as Union for International Cancer Control (UICC) TNM classification (8th edition), cT3‐4 N0 M0 or Tany N+ M0; and (i) preserved organ function. The exclusion criteria were (i) undergoing major surgery, radiation therapy, or prior chemotherapy within 4 weeks of study inclusion; (ii) a history of severe lung disease; (iii) with a stent for stenosis; (iv) hepatitis B surface (HBs) antigen or hepatitis C virus (HCV) antibody positivity; (v) serious comorbidities (heart failure, renal failure, liver failure, hemorrhagic peptic ulcer, intestinal paralysis, intestinal obstruction, uncontrolled diabetes, etc.); (vi) active multiple cancers (simultaneous multiple cancers or metachronous multiple cancers with a disease‐free period of ≤5 y); and (vii) pregnancy or breastfeeding. The complete inclusion and exclusion criteria are provided in Data S1.

### 2.2 Treatment

After registration, each patient received LCCRT (1.8 Gy × 28 fractions; total 50.4 Gy) concurrent with capecitabine (capecitabine 1600 mg/m^2^/day orally twice daily on the day of irradiation) and CNCT (four courses of CAPOX; capecitabine 2000 mg/m^2^ orally twice daily on Days 1–14, oxaliplatin 130 mg/m^2^ intravenously on Day 1, every 3 weeks) followed by total mesorectal excision (TME) or tumor‐specific mesorectal excision (TSME) (Figure 1). The radiation field encompassed the tumor bed with a margin, in addition to regional lymph nodes according to tumor location and growth area. The mesorectal, presacral lymph nodes, lateral obturator nodes, and internal iliac nodes were consistently included. In cases where the primary tumor invaded the bladder, prostate, cervix, or vagina, the external iliac nodes should be included. Both 3D conformal radiation therapy and intensity‐modulated radiation therapy (IMRT) were available. The protocol stipulated that CAPOX should be initiated 2–4 weeks after LCCRT completion. If treatment could not be started because of an adverse event, CNCT was delayed for up to 5 weeks. Surgery was performed 3–8 weeks after the last dose of CAPOX (last day of capecitabine administration) or on the date of discontinuation and included low anterior resection (LAR), intersphincteric resection (ISR), abdominoperineal resection (APR), and Hartmann operation. For patients with suspected invasion of adjacent organs, combined resection of adjacent organs was also acceptable to achieve radical resection. Additional LLND was acceptable at the surgeon's discretion. The surgical approach (laparotomy, laparoscopy, or robot‐assisted surgery) was not specified.

Each patient underwent tumor restaging to clinical complete response (cCR), near‐complete response (nCR), and incomplete clinical response (iCR) based on colonoscopy, pelvic magnetic resonance imaging (MRI), and digital findings according to the Memorial Sloan Kettering Cancer Center (MSKCC) Regression Schema^16^ within 1–3 weeks after CAPOX completion (last day of capecitabine administration) or the date of discontinuation at each of the participating institutions. The clinical response rate based on pelvic MRI was also assessed using the Response Evaluation Criteria in Solid Tumors (RECIST) v1.1. Nonoperative management (NOM) was allowed if cCR or nCR was obtained during preoperative restaging and the patient requested NOM.

### 2.3 Pathological analysis

Although appropriate evaluation criteria have not yet been defined, the American Joint Committee on Cancer (AJCC) evaluation method is the most widely used and internationally prognostically relevant. Briefly, the AJCC tumor regression grading (TRG) was classified into four histological TRGs based on vital tumor tissue at the ratio of fibrosis: TRG 0, complete regression and the absence of viable cancer cells; TRG 1, only small clusters or single cancer cells; TRG 2, residual cancer cells but with predominant fibrosis; and TRG 3, minimal or no decrease in tumor cells or extensive residual cancer.^17^ This method was also used to determine the pCR in this study. pCR was defined as the absence of viable tumor cells in both the primary tumor and lymph nodes (ypT0N0).

### 2.4 Follow‐up

Follow‐up was performed every 3 mo for the first 3 y and every 6 mo thereafter for up to 5 y. The levels of the tumor markers carcinoembryonic antigen (CEA) and carbohydrate antigen 19–9 (CA19‐9) were assessed at each follow‐up examination. Chest‐abdominal‐pelvic CT was performed every 6 mo. Total colonoscopies were performed annually. For patients with NOM, tumor marker testing, colonoscopy, rectal examination, pelvic MRI, and chest‐abdominal‐pelvic CT every 4 mo were recommended in the first 2 y and every 6 mo thereafter for up to 5 y.

### 2.5 Endpoints and statistical analysis

The primary endpoint was the pCR rate. The secondary endpoints were the cCR rate, R0 resection rate, and safety in terms of adverse events, relapse‐free survival, overall survival, and recurrence pattern (local and distant recurrence rates).

In accordance with ENSEMBLE‐1,^15^ the pCR rate in previous phase III trials in which SCRT was followed by surgical treatment was 0.5%–1%. With reference to these results and the fact that TNT was added in this trial, the present trial also examined whether 5% could be rejected. The expected pCR rate was set at 30% for patients treated with TNT followed by surgery, as the expected value of the study treatment (RAPIDO).^9^ With an expected pCR rate of 30% and a ± 15% precision of 90% confidence interval (CI) based on the modified Wald method, the sample size was set at 27 patients.

The pCR rate and 90% CI were estimated for the surgical population that received at least one dose of the protocol therapy, met all selection criteria, did not violate any exclusion criteria, and underwent the surgery. The pCR + sustained cCR rate was calculated for the full analysis set, which included both the primary analysis population and nonsurgical population. The overall frequencies and percentages were summarized for the demographic and clinicopathological characteristics. All statistical analyses were performed using SAS v. 9.4 (SAS Institute, Cary, NC, USA) and GraphPad Prism v. 6.01 for Windows (GraphPad Software, San Diego, CA, USA).

## 3 RESULTS

**FIGURE 2:**
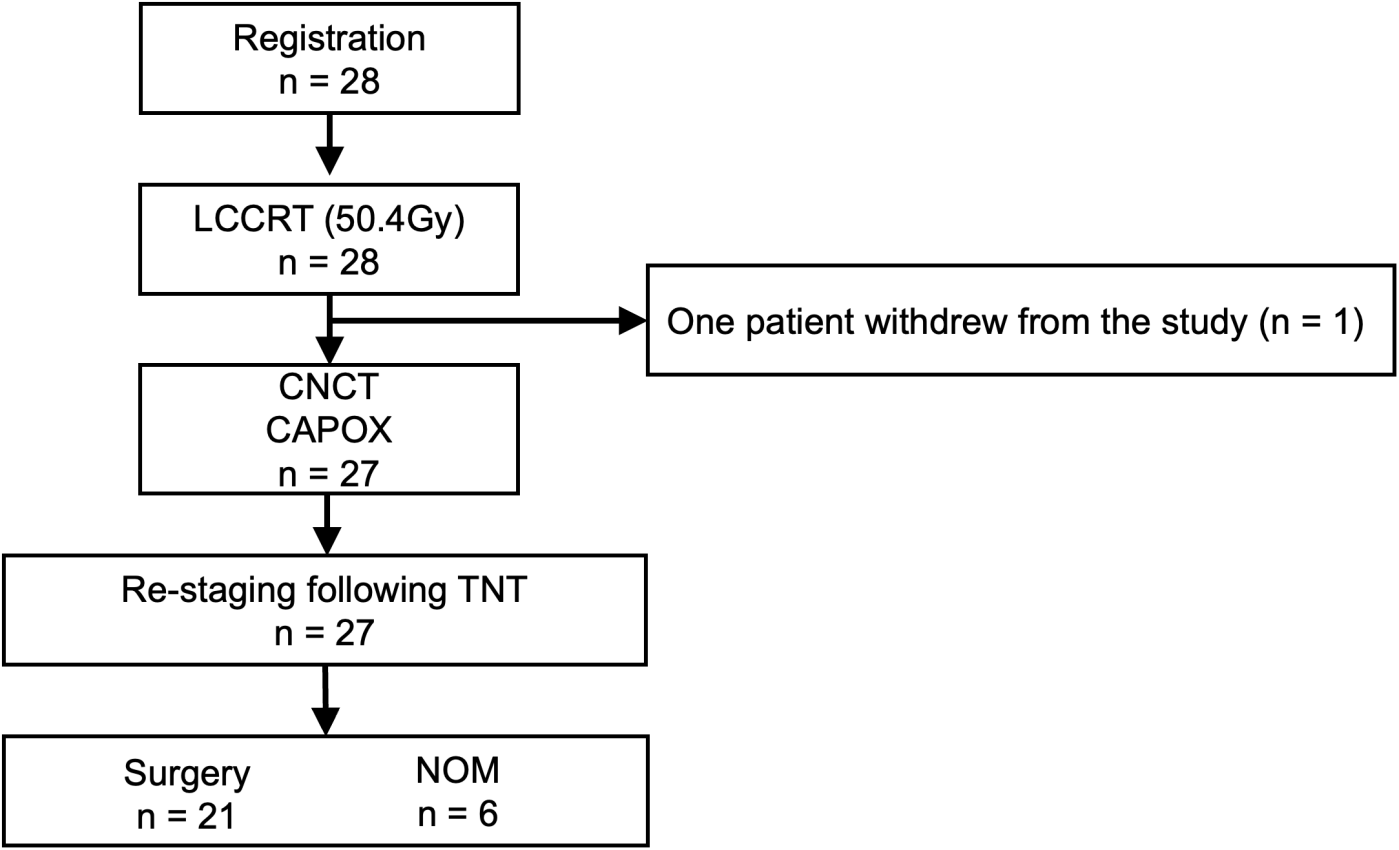
CONsolidated Standards Of Reporting Trials (CONSORT) diagram. The study enrolled a total of 28 patients. One patient withdrew from the study after completing long‐course chemoradiotherapy (LCCRT). Twenty‐seven patients received consolidative chemotherapy (CNCT). Of the remaining 27 patients, 21 underwent total mesorectal excision (TME) and six underwent nonoperative management (NOM). CRT, chemoradiotherapy; TNT, total neoadjuvant treatment; TME, total mesorectal excision; NOM, nonoperative management.

This study enrolled 28 patients with LARC managed at 10 institutions between April and December 2022. Figure 2 shows the flow diagram illustrating patient enrollment and progression through the study protocol. The data were collected up to the end of December 2023, and a median follow‐up of 14.8 (7.3–17.7) mo was observed between the enrollment and the data cutoff points.

### 3.1 Patient characteristics

The clinical characteristics of the 28 enrolled patients are summarized in Table 1. The median patient age was 69.5 y (range, 41–79 y), and 19 (67.9%) patients were male. The median pretreatment CEA was 2.9 (range, 0.4–144) ng/mL. The median tumor diameter was 40 (range, 20–70) mm. The median distance from the tumor to the AV was 43 (range, 0–110) mm. The tumor location was the upper rectum in five patients (17.9%) and the lower rectum in 23 (82.1%) patients. The tumor depth was cT3 in 24 patients (85.7%) and cT4a in four patients (14.3%). Lymph node metastasis was classified as cN0 in 15 patients (53.6%), cN1 in eight patients (28.5%), and cN2 in five patients (17.8%). The UICC TNM stage was cStage II in 15 patients (53.6%) and cStage III in 13 (46.4%) patients. Regarding genomic status, wildtype *RAS* was detected in 10 patients (35.7%), wildtype *BRAF* was detected in 28 patients (100%), and microsatellite instability high (MSI‐H) was detected in one patient (3.6%).

**TABLE 1:** Patient characteristics at enrollment.

Variables	*n* = 28
Age (y) (range)	69.5 (41–79)
Male/Female	19 (67.9%)/9 (32.1%)
BMI (kg/m^2^) (range)	22.6 (16.3–35.6)
Pretreatment CEA (ng/mL) (range)	2.9 (0.4–144)
Pretreatment Hb (g/dL) (range)	13.7 (8.4–15.6)
Tumor size (mm) (range)	40 (20–70)
Tumor location AV (mm) (range)	43 (0–110)
Tumor location upper / lower	5 (17.9%)/23 (82.1%)
cT	
cT1	0
cT2	0
cT3	24 (85.7%)
cT4a	4 (14.3%)
cT4b	0
cN	
cN0	15 (53.6%)
cN1a	2 (7.1%)
cN1b	6 (21.4%)
cN2a	3 (10.7%)
cN2b	2 (7.1%)
Genomic status	
*RAS* wildtype/mutant	10 (35.7%)/18 (64.3%)
*BRAF* wildtype/mutant	28 (100%)/0 (0%)
MSS/MSI‐high/unknown	25 (89.3%)/1 (3.6%)/2 (7.1%)

*Note*: Data are presented as median (range) or number (%).

Abbreviations: AV, anal verge; BMI, body mass index; CEA, carcinoembryonic antigen; Hb, hemoglobin; MSI, microsatellite instability; MSS, microsatellite stable.

### 3.2 Compliance of TNT

All patients received and completed LCCRT (100%). The median treatment duration was 40 d (36–50 d). No patients discontinued treatment because of adverse events. One patient withdrew consent before CNCT. Twenty‐seven patients underwent CNCT at a median of 22 d (range, 14–53 d) after radiotherapy completion. CNCT initiation was postponed in three patients because of adverse events associated with LCCRT (diarrhea). Twenty‐seven patients (96.4%) completed four courses of CAPOX. TNT was completed in 27 patients (96.4%) (Figure 2 and Table 2).

**TABLE 2:** Outcomes of preoperative treatment and surgery.

Variables	*n* = 28
LCCRT completion	28 (100%)
CAPOX completion	27 (96.4%)
Completion of protocol treatment including TME	21 (75.0%)
Restaging (MSKCC regression schema)	
cCR/nCR/iCR/NE treatment after TNT	10 (35.7%)/8 (28.6%)/9 (32.1%)/1 (3.6%)
TME	21 (75.0%)
NOM	6 (21.4%)
Others^a^	1 (3.6%)

Surgical outcomes	*n* = 21
LAR/ISR/APR or Hartman	10 (47.6%)/5 (23.8%)/6 (21%)
With LLND/with diverting stoma	3 (14.3%)/21 (100%)
Operation time (min) (range)	397 (220–698)
Estimated blood loss (mL) (range)	140 (0–337)
Conversion to open surgery	0
Curative resection (Cur A/Cur B)	18 (85.7%)/3 (14.3%)
Postoperative complication (CD ≥3)	
Abdominal abscess	1 (4.7%)
Postoperative hospital stay (d) (range)	13 (6–24)
Postoperative mortality	0
Postoperative recurrence^a^, ^b^	3 (10.7%; lung 1, local 1, peritoneum 1)

*Note*: Data are presented as median (range) or number (%).

Abbreviations: APR, abdominoperineal resection; cCR, clinical complete response; CD, Clavien–Dindo classification; iCR, incomplete response; ISR, intersphincteric resection; LAR, low anterior resection; LCCRT, long‐course chemoradiotherapy; TME, total mesorectal excision; LLND, lateral pelvic lymph node dissection; nCR, near‐complete response; NE, not evaluable; TNT, total neoadjuvant therapy.

^a^ Lost to follow‐up because of patient's request.

^b^ Median follow‐up: 14.8 (range: 10.5–22.8) mo.

### 3.3 Adverse events during TNT

Adverse events related to LCCRT and CNCT are shown in Table 3. The most common LCCRT‐related adverse event of any grade was diarrhea (28.6%). The rates of CNCT‐related adverse events of grade ≥3 were as follows: diarrhea, 7.1%; neutropenia, 7.1%; and peripheral neuropathy, 3.6%. There were no treatment‐related deaths.

**TABLE 3:** Adverse events during TNT (CTCAE v. 5.0).

Adverse events during TNT	*n* = 28	Grade 1	Grade 2	Grade 3	Grade 4
Adverse events during CRT					
Diarrhea		8 (28.6%)	0 (0.0%)	1 (3.6%)	0 (0.0%)
Adverse events during CAPOX therapy					
Nausea/vomiting		9 (32.1%)	2 (7.1%)	0 (0.0%)	0 (0.0%)
Anorexia		5 (17.9%)	4 (14.3%)	0 (0.0%)	0 (0.0%)
Diarrhea		7 (25.0%)	1 (3.6%)	2 (7.1%)	0 (0.0%)
Constipation		5 (17.9%)	1 (3.6%)	1 (3.6%)	0 (0.0%)
Elevation of AST or ALT		12 (42.9%)	1 (3.6%)	0 (0.0%)	0 (0.0%)
Fatigue		6 (21.4%)	2 (7.1%)	0 (0.0%)	0 (0.0%)
Hand‐foot syndrome		8 (28.6%)	2 (7.1%)	1 (3.6%)	0 (0.0%)
Peripheral neuropathy		18 (64.3%)	6 (21.4%)	1 (3.6%)	0 (0.0%)
Skin disorders		0 (0.0%)	0 (0.0%)	0 (0.0%)	0 (0.0%)
Melena		3 (10.7%)	0 (0.0%)	0 (0.0%)	0 (0.0%)
Leukopenia		2 (7.1%)	13 (46.4%)	2 (7.1%)	0 (0.0%)
Neutropenia		1 (3.6%)	10 (35.7%)	1 (3.6%)	0 (0.0%)
Thrombocytopenia		0 (0.0%)	1 (3.6%)	0 (0.0%)	0 (0.0%)
Anemia		8 (28.6%)	5 (17.9%)	0 (0.0%)	0 (0.0%)
Renal dysfunction		5 (17.9%)	0 (0.0%)	0 (0.0%)	0 (0.0%)
Stomatitis or taste disorders		2 (7.1%)	1 (3.6%)	0 (0.0%)	0 (0.0%)
Treatment‐related death	0 (0.0%)				

*Note*: Data are presented as number (%).

Abbreviations: ALT, alanine aminotransferase; AST, aspartate aminotransferase; CRT, chemoradiotherapy.

### 3.4 Restaging after TNT

Twenty‐seven patients (96.4%) underwent restaging for efficacy using MRI, colonoscopy, and a digital examination. MRI was performed at a median of 14 d (range, 0–60 d) after the last chemotherapy dose or the decision to discontinue chemotherapy and at a median of 122.5 d (range, 98–180 d) after the start of protocol treatment. The clinical responses and rates were cCR (35.7%, 10/28 patients), nCR (28.6%, 8/28), iCR (32.1%, 9/28), and not evaluable (NE) (3.6%, 1/28). One patient (3.6%) developed lung metastasis during TNT.

### 3.5 Treatment after TNT

Twenty‐one patients underwent TME (66.7%). The surgical approach was robotic in 13 patients (61.9%) and laparoscopic in eight patients (38.1%). No patients required conversion to open surgery; thus, all patients (100%) underwent minimally invasive surgery. LAR, ISR, and APR or Hartman's procedure were performed in 10 (47.6%), five (28.3%), and two (9.5%) patients, respectively. LLND was performed in three (14.3%) patients. A diverting stoma was created in all 21 patients (100%) who underwent TME. Curative resection (CurA) was performed in 18 patients (85.7%). Three patients were classified as having CurB: one with pathologic RM1, one with pathologic DM1, and one with lung metastasis. The patient with lung metastasis underwent simultaneous resection of the primary tumor and the metastasis. One patient (4.7%) exhibited a Clavien–Dindo grade 3 abdominal abscess. The median postoperative hospital stay was 13 d (range, 6–24 d) (Table 2). No patients died.

### 3.6 Pathological findings

The pathological findings of the patients who underwent surgery are shown in Table 4. According to TRG classification, 23.8%, 14.3%, 42.9%, and 14.3% of cases were TRG 0, 1, 2, and 3, respectively. The pCR rate, the primary endpoint, was 23.8% (90% confidence interval [CI] 11.8%–41.8%), and the final classification included ypStage 0 (five patients, 23.8%), I (seven patients, 33.3%), II (seven patients, 33.3%), III (one patient, 4.8%), and IV (one patient, 4.8%). One patient developed lung metastasis during TNT. The downstaging rate in terms of T stage was 66.7% (14/21 patients). All cT4 tumors were downstaged after TNT. The downstaging rate in terms of N stage was 47.6% (10/21 patients).

**TABLE 4:** Pathological findings of the resected specimens.

Variables	*n* = 21
Histological type	
tub1/tub2/no findings	5 (23.8%)/11 (52.4%)/5 (23.8%)
Macroscopic classification	
Type 2/5/no findings/others	9 (42.9%)/7 (33.3%)/3 (14.3%)/2 (9.5%)
Tumor regression grade	
TRG0/TRG1/TRG2/TRG3	5 (23.8%)/3 (14.3%)/10 (47.6%)/3 (14.3%)
ypT	
ypT0	5 (23.8%)
ypT1	2 (9.5%)
ypT2	6 (28.6%)
ypT3	6 (28.6%)
ypT4a	1 (4.8%)
ypT4b	1 (4.8%)
ypN	
ypN0	20 (94.2%)
ypN1a	0
ypN1b	1 (4.8%)
ypN2	0
Pathologically complete response	5 (23.8%)
Number of lymph nodes resected (range)	6 (0–32)
Positive lymphatic invasion	0
Positive vascular invasion	6 (28.6%)
Positive tumor budding (BD)	2 (9.5%)
Positive neural invasion (Pn)	6 (28.6%)
Positive extramural cancer deposits (EX)	0

*Note*: Data are presented as median (range) or number (%).

Abbreviations: tub1, well differentiated tubular adenocarcinoma; tub2, moderately differentiated tubular adenocarcinoma.

### 3.7 NOM following TNT

Six patients (21.4%) opted for NOM, including five with cCR and one with nCR. The median follow‐up period for the NOM cohort was 15.5 mo (range, 10.5–17.5 mo). All patients underwent at least two rounds of screening, including digital examination, MRI, colonoscopy, and CT. No regrowth was observed. pCR + sustained cCR was observed in 10 out of 28 patients, equating to a rate of 35.7% (90% CI: 22.6%–51.3%). The rate of pCR + NOM was 39.3% (90% CI: 25.7%–54.8%).

## 4 DISCUSSION

This is the first multicenter phase II clinical trial in Japan to evaluate the feasibility and safety of TNT consisting of LCCRT followed by CAPOX in patients with LARC.

The results of the ENSEMBLE‐2 study confirmed that the rates of 23.8%, 35.7%, and 39.3% for pCR, cCR, and pCR + NOM, respectively, were comparable with those observed previously. Previous TNT trials reported pCR rates of TNT of 28.3% (RAPIDO), 27.8% (PRODIGE‐23), and 22.5% (STELLAR).^9^, ^10^, ^11^ In the OPRA trial, the cCR and nCR rates at TNT restating were 41.1% and 37.5%, respectively, while the rate of NOM at 3 y was 47% in the entire cohort.^18^, ^19^

The findings of the ENSEMBLE‐2 study indicated that patients who received LCCRT followed by four courses of CAPOX exhibited good tolerance, with a 96.4% completion rate. However, a higher prevalence of grade 3 toxicity (32.1%) was observed. Pivotal randomized controlled trials demonstrated favorable compliance rates (~85%). Nevertheless, these trials also indicated higher rates of grade ≥3 toxicity, which was observed in 47.6% patients in the RAPIDO trial, 46.9% patients in the PRODIGE‐23 trial, and 26.5% patients in the STELLAR trial.^9^, ^10^, ^11^ Our findings indicate that the efficacy and tolerability of LCCRT followed by CNCT is at least equivalent to those in the setting of neoadjuvant treatment for patients with LARC.

All 21 patients who underwent surgery exhibited CurA or CurB, with no severe surgical complications except one abdominal abscess (3.7%), while R1 resection was diagnosed pathologically as RM1 and DM1 in two patients (9.5%). Previous studies reported R1 resection rates of 10% (RAPIDO), 5% (PRODIGE‐23), and 8.1% (STELLAR) following TNT.^9^, ^10^, ^11^ The feasibility and safety of surgical resection of LCCRT followed by CNCT in Japan were demonstrated in the ENSEMBLE‐2 study.

Currently, patients with LARC are increasingly demanding nonsurgical personalized treatment strategies that are safe, improve the quality of life, and are cost‐effective.^18^, ^22^ In the ENSEMBLE‐2 study, six patients (21.4%) with cCR or nCR were treated with NOM in accordance with the clinical trial protocol. Previous randomized studies demonstrated that TNT contributes to not only enhanced disease‐free survival and overall survival but also pCR/cCR.^9^, ^10^, ^11^ The European Society of Medical Oncology (ESMO) guidelines categorize rectal cancer into four distinct stages: early, intermediate, locally advanced, and advanced. TNT is recommended for patients with advanced disease, defined as cT3 with mesorectal fascia involvement, levator‐threatened, positive lateral nodes, or cT4.^20^ The National Comprehensive Cancer Network (NCCN) guidelines (v. 1, 2024) indicate TNT as the standard treatment for LARC with microsatellite stable/proficient mismatch repair (MSS/pMMR). According to the NCCN guidelines, NOM is a potential treatment option for patients with cCR in medical centers with an experienced multidisciplinary team.^21^

The relative efficacy and detrimental effects of SCRT and LCCRT on TNT remain controversial. In the surgical setting, conventional neoadjuvant SCRT and LCCRT are equivalent regarding local control,^23^, ^24^ other oncological endpoints, and patient quality of life.^25^, ^26^ The recently updated results from the RAPIDO trial have prompted a debate regarding the efficacy of SCRT‐CNCT. The results indicated a higher incidence of locoregional failure with SCRT‐CNCT than with conventional CRT.^12^ Previous phase III trials^11^ and meta‐analyses^27^ have demonstrated comparable local control between SCRT‐CNCT and conventional LCCRT. A recent retrospective report from the MSKCC reported a 2‐y organ preservation rate of 40% with LCCRT‐TNT, which was numerically higher than the 2‐y organ preservation rate of 29% with SCRT‐TNT. Among patients with a cCR who opted for NOM, the 2‐y local regrowth rates were 20% with LCCRT‐TNT and 36% with SCRT‐TNT.^28^ To ascertain the efficacy of SCRT‐CNCT and LCCRT‐CNCT in Japan, we plan to use clinical data from ENSEMBLE‐1 and ENSEMBLE‐2 to evaluate the short‐ and long‐term outcomes of these treatment modalities. Considering concerns regarding the durability of local control with SCRT, the ongoing CAO/ARO/AIO‐18.1 trial (NCT04246684) is positioned to definitively address this question. The trial hypothesized that the 3‐y organ preservation rates of LCCRT‐CNCT would be superior to those of SCRT‐CNCT.

Current neoadjuvant treatment options include CRT or radiotherapy alone, chemotherapy alone, and TNT. Moreover, there is ongoing debate regarding the use of SCRT or LCCRT in combination with induction or consolidation chemotherapy, as well as the selection of doublet or triplet chemotherapy regimens in the context of TNT. The international collaboration among the JANUS rectal cancer trial (NCT05610163), ACO/ARO/AIO18.1 (NCT04246684), and ENSEMBLE (jRCTs031220342/NCT05646511)^29^ is currently elucidating relevant clinical questions regarding modern TNT options.^30^

The ENSEMBLE‐2 study has some limitations. First, it included a limited number of participants. Second, the follow‐up period was relatively short because this study was not designed to investigate long‐term prognostic improvement. Instead, it was designed to investigate the feasibility and safety of introducing TNT comprising LCCRT‐CNCT in Japan. Third, the long‐term safety of NOM administration following TNT has not been established and requires further investigation.

In conclusion, the results of this study demonstrated the comparable efficacy, feasibility, and tolerability of TNT, consisting of LCCRT‐CNCT, for Japanese patients with LARC. ENSEMBLE‐2 will investigate long‐term outcomes, MRI analysis, and prediction of the efficacy of circulating tumor DNA via liquid biopsy. Moreover, clinical data from ENSEMBLE‐1 and ENSEMBLE‐2 will be analyzed to assess the efficacy of the treatment, adverse events, safety, and surgical procedures following TNT in Japan.

## AUTHOR CONTRIBUTIONS

Yoshinori Kagawa: Conceptualization; Writing—original draft; formal analysis; Writing—review and editing. Ando Koji: Conceptualization; formal analysis; Writing—review and editing. Mamoru Uemura: Conceptualization; formal analysis; Writing—review and editing. Jun Watanabe: Conceptualization; formal analysis; Writing—review and editing. Koji Oba: Conceptualization; formal analysis; Writing—review and editing. Yasunori Emi: Writing—review and editing. Nobuhisa Matsuhashi: Writing—review and editing. Naoki Izawa: Writing—review and editing. Osamu Muto: Writing—review and editing. Tatsuya Kinjo: Writing—review and editing. Ichiro Takemasa: Writing—review and editing. Eiji Oki: Conceptualization; Writing—original draft; formal analysis; Writing—review and editing.

## FUNDING INFORMATION

This research received no specific grant from any funding agency in the public, commercial, or not‐for‐profit sectors.

## CONFLICT OF INTEREST STATEMENT

Yoshinori Kagawa and Eiji Oki have conflicts of interest in Chugai Pharmaceutical. Nobuhisa Matsuhashi has conflicts of interest in Chugai Pharmaceutical and Yakult Honsha. Naoki Izawa has conflicts of interest in Taiho. Jun Watanabe, Ichiro Takemasa, and Eiji Oki are editorial members of *Annals of Gastroenterological Surgery*. The other authors declare no conflicts of interest for this article.

## ETHICS STATEMENTS

Approval of the study protocol: All procedures performed in this study were in accordance with the approval of the Institutional Review Board of the Kyushu University Certified Review Board (CRB7180005).Informed consent: Written informed consent was obtained from all patients before enrollment.Registry and the Registration No. of study trail: This study was registered in the Japan Registry of Clinical Trials (jRCTs071210143).Animal studies: Not conducted in this study.

## Supporting information


**Data S1:** Supporting Information.

## Data Availability

Dr. Kagawa has full access to all data in the study and takes responsibility for the integrity of the data and the accuracy of the data analysis.
