# Supplementary material for: Phase II study of long‐course chemoradiotherapy followed by consolidation chemotherapy as total neoadjuvant therapy in locally advanced rectal cancer in Japan: ENSEMBLE‐2
Source: Ann Gastroenterol Surg. 2024 Aug 3;8(6):1067–75. doi: 10.1002/ags3.12848 (PMC11533031; doi:10.1002/ags3.12848)
Supplement: Supplementary file 1 — Data S1: Supporting Information. [file AGS3-8-1067-s001.docx]

**Supporting Information**

**Supplement 1: Inclusion/Exclusion Criteria**

**Inclusion Criteria**

1. Each patient was fully informed of the study content, and their written consent was obtained.

2. Histologically confirmed adenocarcinoma of the rectum.

3. No distant metastasis on imaging studies and clinically amenable to curative resection.

4. Age ≥20 years on the date of consent

5. Eastern Cooperative Oncology Group Performance status (ECOG PS) 0 to 1 (ECOG PS 0 if the patient was ≥71 years of age on the date of consent).

6. Prior untreated rectal cancer with the inferior margin of the tumor within 12 cm of the anal verge (AV).

7. Clinically diagnosed as Union for International Cancer Control (UICC) TNM classification (8th edition) cT3-4N0M0 or Tany N+M0 at the time of diagnosis before the start of treatment (a lymph node with a short diameter of ≥10 mm was considered positive).

8. The following criteria for major organ function fulfilled within 14 days before enrollment: If more than one test result existed within the relevant period, the most recent result was used for the measurement. Blood transfusions or hematopoietic factor products were not administered within 14 days of the test date.

a. Neutrophil count: ≥1,500/mm^3^

b. Platelet count: ≥10.0 × 10^4^/mm^3^

c. Hemoglobin concentration: ≥9.0 g/dL

d. Total bilirubin: ≤1.5 times the upper limit of the institutional standard

e. Aspartate aminotransferase (AST), alanine transaminase (ALT), and alkaline phosphatase (ALP) ≤2.5 times the upper limit of the institutional standard (≤5 times in patients with liver metastasis)

f. Serum creatinine: ≤1.5 times the upper limit of the institutional standard, or creatinine clearance ≥45 mL/min.

**Exclusion Criteria**

1. Patients received any of the following treatments within a certain time before the start of protocol therapy:

a. Extensive surgery within 4 weeks (excluding central venous [CV] port placement and stoma creation)

b. Any anticancer therapy within 4 weeks

c. Radiation within 4 weeks

2. Concomitant or preexisting severe pulmonary disease (interstitial pneumonia, pulmonary fibrosis, severe emphysema)

3. Patients with implanted colorectal stents

4. Serious comorbidities (heart failure, renal failure, liver failure, bleeding peptic ulcer, intestinal paralysis, bowel obstruction, and poorly controlled diabetes)

5. Patients with active multiple overlapping cancers (synchronous multiple overlapping cancers or iatrogenic multiple overlapping cancers with a disease-free period of ≤5 years). However, carcinoma in situ (intraepithelial carcinoma) or intramucosal carcinoma that was considered curable via local treatment was not considered active multiple overlapping carcinoma

6. Pregnant or lactating women, positive pregnancy test or unwillingness to use contraception

7. Hepatitis B surface (HBs) antigen or hepatitis C virus (HCV) antibody positivity

8. Known human immunodeficiency virus (HIV) infection

9. Judged by the principal investigator or subinvestigator to be unsuitable for this study.
